# Characteristics of the Washington cannabis market from 2014 to 2016

**DOI:** 10.1186/s42238-022-00147-8

**Published:** 2022-07-04

**Authors:** William C. Kerr, Yu Ye

**Affiliations:** Alcohol Research Group, Public Health Institute, 6001 Shellmound Ave, Suite 450, Emeryville, CA 94608 USA

**Keywords:** Cannabis, Marijuana, Market share, Legal, Illegal, Washington State, Marijuana products

## Abstract

**Background:**

The state of Washington legalized cannabis for adult use in 2012 and retail stores began to open in 2014. While details of the legal market have been tracked by the state, the total market for cannabis and characteristics of purchasers can only be identified through surveys.

**Methods:**

Six cross-sectional samples of the Privatization of Spirits in Washington (PSW) surveys were collected between January 2014 and October 2016 with two in each year. Random digit dial procedures were used to recruit a sample aged 18 and older, with 40% of cases from mobile phones. A total of 5492 respondents participated. Analyses of the population-weighted sample utilized purchasing amounts and frequencies, use frequency and related measures to estimate total and mean amounts and expenditures. Sensitivity analyses were conducted for key assumptions.

**Results:**

The market for cannabis flower is estimated to have increased from 158 metric tons and $1.23 billion in 2014 to 222 metric tons and $1.7 billion in 2016, with little change from 2014 to 2015. Purchases from legal sources, retail and dispensaries, were estimated at 69% of the total market. Daily or near daily (DND) users accounted for about 83% of sales in 2014, rising to 91% in 2016. The prevalence of past year use rose substantially from 25% in 2014 to 32% in 2016, with DND use rising from 10.2 to 11.3%. Average purchase amounts for DND users rose from 291 g in 2014 to 374 g in 2016, while mean amounts among non-DND users declined from 78 to 28.6 g.

**Conclusions:**

The expansion of retail cannabis stores in Washington appears to have led to increased market size in 2016 with all of the increase attributed to DND users. Frequent users may be important to consider in legalization evaluations.

## Background

With cannabis for adult use becoming legal in an increasing number of states, issues related to the transition from illicit to legal cannabis markets are a significant research priority. Key questions regarding the impacts of cannabis legalization include the degree to which purchasing will shift from the illicit market and the how the market will grow and change. Estimating the size of illicit markets is difficult, but general population surveys offer what is possibly the best opportunity for this in states where cannabis use is legal and where cannabis can be purchased in retail stores. Prior studies have utilized frequency of use data from the National Surveys on Drug Use and Health (NSDUH) series along with data from other sources to estimate use amounts and expenditures (Caulkins et al. 2019) and data on purchasing in the NSDUH to estimate sales and other aspects of how marijuana is obtained in the USA (Davenport and Caulkins 2016).

National trends in cannabis use show a steady increase in past year use prevalence among those 12 and older from 10.1% in 2007 to 15.9% in 2018, with a similar increase in past month use (Substance Abuse and Mental Health Services Administration 2018). In a national study of marijuana trends utilizing age-period-cohort decomposition in models with sociodemographic and cannabis policy measures to identify sources of marijuana use prevalence variation over the 1984–2015 period, it was found that the increased use rates through 2015 could be attributed to general period effects rather than specific state policy changes (Kerr et al. 2018). However, few states had adopted legalization in that period and the varying details of medical use laws make those difficult to evaluate. Nonetheless, it appears that more general changes in attitudes regarding marijuana was an important factor (Stringer and Maggard 2016).

Legalization in the state of Washington was passed in 2012 but retail stores did not open until July of 2014, with 31 stores open by the end of August, then rising to 311 stores open as of September 2016. Washington already allowed medical use for a wide range of conditions with dispensary sales and had an established illicit market with high prevalence of use (Kerr et al. 2018). Washington adult use cannabis market regulations included restrictions on store numbers, seed to sale tracing, bans on home growing and delivery, and relatively high tax rates, although taxes were simplified and reduced in 2015 (Cambron et al. 2017). Further restrictions including temporary and permanent bans on stores and location restrictions were also in place at local levels (Dilley et al. 2017).

Prior analyses from the same Washington survey series utilized here have provided insights into changes in cannabis use and related attitudes during the early years of the legal retail market. Use prevalence did not substantially increase with adult use legalization from 2012 to 2014 and 2015 (Kerr et al. 2018), but, in 2016 use prevalence did increase significantly (Subbaraman and Kerr 2020). Other results from the same survey series found that prevalence rates of marijuana harms from others’ use were flat from 2014 to 2016 (Kerr et al. 2021) and that variation in individual’s alcohol and cannabis use in the linked longitudinal sample indicated that more frequent cannabis use was tied to risky drinking (Kerr et al. 2019). Furthermore, voters support for legalization was shown to increase after implementation among those who voted both for and against I502, the initiative establishing legal adult use (Subbaraman and Kerr 2016), and population support for cannabis legalization in Washington continued to increase to 78% in 2016 (Subbaraman and Kerr 2017).

Studies of the Washington market have found increased use over time after adult use legalization. A study utilizing analyses of raw wastewater sample from 2014 to 2016 found evidence of increasing use in 2016, consistent with survey results (Burgard et al. 2019). A study utilizing the Behavioral Risk Factor Surveillance System survey for Washington found increased use and frequent use after legalization to be associated with access to retail stores, rather than legalization or store opening generally (Everson et al. 2019). The details of cannabis purchases in the legal market from 2014 to 2016 have also been evaluated finding that the purchases mostly involved high-THC cannabis flower with a growing share of cannabis extracts and a declining price per gram, particularly from 2014 to 2015 (Smart et al. 2017).

The current study presents estimates of cannabis market trends in purchasing, purchase frequency, amounts, and types and total market size for Washington from 2014 to 2016 as retail stores opened. These estimates are relevant to understanding the early development of cannabis markets after legalization and store openings and for considering the implications of legalization on illicit markets and population use patterns. Highlighting user and purchaser characteristics related to more frequent use, large purchase amounts, and providing cannabis to others are also needed for developing and targeting prevention and harm reduction efforts.

## Methods

### Sample

The series of Privatization of Spirits in Washington (PSW) Surveys, conducted between January 2014 and December 2016 by ICF International, was designed to evaluate impacts over time of the privatization of spirits sales and the legalization of marijuana in Washington state. The data analyzed consist of six cross-sectional representative surveys of adults (aged 18 and over), with sample recruitment taking place separately in January–April 2014 (Wave 1, *N* = 1202), September–October 2014 (Wave 2, *N* = 804), March–May 2015 (Wave 3, *N* = 823), August–October 2015 (Wave 4, *N* = 662), March–April 2016 (Wave 5, *N* = 610), and September–December 2016 (Wave 6, *N* = 1391). At each wave, respondents were selected using a state random probability sample obtained via random digit dial (RDD) of both landline and cell phone samples with about 40% cell phones. Respondent self-identified as adult Washington residents. The AAPOR2 cooperation rates (The American Association for Public Opinion Research 2011) (complete and partial interviews as a percentage of identified eligible respondents) were (landline, cell): Wave 1 (50.8%, 59.5%), Wave 2 (45.8%, 62.4%), Wave 3 (43.7%, 61.5%), Wave 4 (41.7%, 59.6%), Wave 5 (49.4%, 60.9%), and Wave 6 (45.3%, 63.0%). Surveys lasted about half an hour on average and respondents received $10 gift cards. Protocols were approved by the Public Health Institute Institutional Review Board (#I13-010).

### Measures

#### Past-year (PY) marijuana user

*Past-year (PY) marijuana user* was determined using the question: “How often have you used marijuana, hash or pot during the last twelve months,” with selection options including “Every day or nearly every day,” “About once a week,” “Once every 2 or 3 weeks,” “Once every month or two,” “less often than that,” and “Never last year.” Respondents were coded as dichotomous PY marijuana users or not. PY use was further classified into *Daily or Nearly Daily (DND) users* (those who answered “every day or nearly every day”) and *non-DND users*. *Mode of marijuana use* was elicited by the question “how do you most commonly consume marijuana” and categorized as smoke, inhalable (vaporize marijuana, hashish, resin, oil, wax, or dabs), edible (food product or beverage), and other product (tincture, lotion, salve, balm, spray, or other).

#### PY flower (raw marijuana) purchasing quantity and expenditure

All PY marijuana users were asked “how often do you usually purchase marijuana” with categorical responses recoded as the *number of days purchased PY*. *Marijuana flower amount (in grams) usually purchased* was based on the question: “what amount of raw Marijuana do you usually buy”, with selection options ranging from “0.5 g (Nickel Bag)” to “an ounce (28 g),” and including “Other: Specify” where respondents provide the specific amount. To elicit expenditure, purchasers were then asked “about how much this amount cost,” and provided the dollar amount for the usual marijuana flower purchase. *Average $ per gram of marijuana flower* was derived dividing usual expenditure on usual flower purchase quantity. Cleaning of expenditure data involved editing noticeable typos and adjusting for potential outliers by truncating average $ per gram at the 5th and 95th percentiles. Across six waves there were 1177 PY marijuana users*,* and 769 reported purchasing at least once PY. Among them, 59 reported “Never” for usual purchase quantity without valid usual expenditure. They were excluded from the final definition of *flower purchaser* (*n* = 710). *Total quantity (in grams) of marijuana flower purchased PY* was derived multiplying the usual grams of marijuana flower purchased by the number of days marijuana was purchased, assuming each trip involved a marijuana flower purchase. In a sensitivity analysis, we adjusted for potential overestimation by accounting for trips purchasing other marijuana products (described below). Fifty-two respondents reported valid usual expenditure but were missing usual purchase quantity. Their usual quantity was imputed by dividing usual expenditure by the sample median $ per gram. For those who reported usual quantity but were missing on expenditure (*n* = 45 across six waves), their usual expenditure was imputed by multiplying usual quantity by sample median $ per gram as well. Finally, 16 flower purchasers missing both purchase usual quantity and expenditure were assigned the sample median on both measures. *Total $ expenditure on marijuana flower PY* was then calculated multiplying the usual expenditure on flower by the number of days marijuana was purchased.

#### Other marijuana product purchases

All PY marijuana users were asked “how often do you usually purchase marijuana-related products such as hash, oil, edibles, teas or lotions” and coded as *PY purchaser of other marijuana products* or not and *number of days other marijuana products were purchased*. Each purchaser was then asked to provide the *amount usually bought and how much that amount costs* for up to three products. Across six waves there were 391 PY other marijuana product purchasers of whom 240 reported usual expenditure for one product, 64 for two products, and 38 for three products. For those purchasing more than one product, we estimated the usual expenditure assuming that half of the time the products were purchased separately and the other half of the time the products were purchased together. 49 respondents who were other marijuana product purchasers but were missing on usual expenditure were assigned the sample median. Sensitivity analyses also considered the average (assuming all products purchased separately with equal probability) and the summation (assuming all products purchased together) of costs across products. *PY total expenditure on other marijuana products* was calculated from the usual expenditure and number of purchase days.

#### Purchase at a legal retail store (Wave 2–6)

From Wave 2 (no stores had opened at Wave 1), each marijuana user was asked “Since July 2014, have you purchased marijuana, or other marijuana products from a legal retail store in Washington.” For the Wave 5 and 6 surveys in 2016, three items were added for marijuana users: “Within last 12 months, what proportion of your marijuana purchases were from…”: “a legal store in Washington,” “a medical dispensary in Washington,” and “other sources” with options of “none,” “less than half,” “about half,” “most,” or “all.” These responses were converted to proportions (i.e., 0%, 25%, 50%, 75%, and 100%, respectively), multiplied by self-reported purchasing quantity and expenditure on marijuana flower to estimate the flower market size from *store sales* (i.e., sales from both legal retail stores and medical dispensaries) and from the illegal market (i.e. sales from “other sources”) in 2016.

### Statistical analyses

The two cross-sectional waves in each year were combined to generate data for years 2014, 2015, and 2016 separately. Merging each two waves adjusts for potential seasonal fluctuations in marijuana use and purchase behavior, and enhances stability with larger sample size. Sample proportions and means for population prevalence and average estimates are presented. To test trend effects, regressions with survey year as a continuous predictor were estimated. Marijuana market size estimates aggregated individual quantities applying the population weights. All analyses were performed with STATA version 15 survey commands (StataCorp. 2017).

The data were weighted to adjust for probability of selection introduced during the sampling design and also to post-stratify and adjust the sample to match the target population. First, base weights were constructed for landline and cell phone samples separately to reflect the number of phones and number of adults for each household (landline sample) or individual number of phones (cell sample) Second, the landline and cell sample were combined to reflect the population coverage of landline and cell sample frames. The respondents were weighted to National Health Interview Survey state benchmarks based on their landline/cell usage status. Last, the weighted data were calibrated to reflect population distributions from the American Community Survey, using a raking adjustment for the following dimensions: sex by age, age by race/ethnicity, and education levels. For the combined cross-section data for 2014, 2015, and 2016 separately, the final weighted sample represents all adults (18 and older) residing in the State of Washington in the given year.

Several sensitivity analyses were performed. First, to adjust for potential overestimation of the marijuana flower market size, since purchasing frequency of marijuana flower was not specifically asked, the number of days marijuana flower was purchased was re-derived for PY marijuana users whose most commonly used product was not a smoked product (i.e., inhalable, edible, or others). Their flower purchasing frequency was re-estimated by taking the larger value of (1) the difference between the number of days marijuana was purchased and the number of days other marijuana products were purchased or (2) half of the days marijuana was purchased. Furthermore, we re-estimated the market size for other marijuana products, re-calculating the usual expenditure as the average and summation when the respondents reported more than one product was usually purchased.

## Results

Table 1 shows the prevalence of PY marijuana use and purchases of flower and other marijuana products by Washington residents for 2014–2016. Prevalence of PY marijuana use increased from 24.9% in 2014 to 31.7% in 2016 among those aged 18 + . No significant trend was observed for DND use, however, increasing slightly from 10.2 to 11.3%. Among PY marijuana users the most commonly used product type was smoked, which significantly decreased over time (80.1% in 2014 to 72.9% in 2016). For the three years combined, DND users were more likely to report smoked (79.8% vs 71.2% among non-DND users) and inhalable (15.9% vs 9.7%) as their most commonly used product, and less likely to use edibles (3.4% vs 16.2%) (data not shown). A significant increase in prevalence of PY marijuana flower purchases was also observed (14.9% to 21.8% 2014–2016). Similarly, PY purchases of other marijuana products increased from 8.0% in 2014 to 13.0% in 2016. In 2014 at Wave 2, only 23.4% of PY users had purchased at a legal retail store, increasing substantially to 65.1% in 2016.

**Table 1:** Trends in marijuana use and purchasing for Washington state from 2014 to 2016

	**2014**	**2015**	**2016**	**Trend test** ^a^
Past year (PY) users—any marijuana use	24.9%	26.2%	31.7%	***P* = 0.002**
Daily/nearly daily (DND) marijuana user	10.2%	10.2%	11.3%	*P* = 0.514
DND user among PY users	41.1%	38.9%	35.7%	*P* = 0.252
Most common product type used^b^				
Smoked	80.9%	70.7%	72.9%	***P* = 0.049**
Inhalable	9.5%	14.4%	12.3%	*P* = 0.387
Edible	8.2%	13.2%	11.9%	*P* = 0.159
Others	1.4%	1.8%	2.9%	*P* = 0.192
Any marijuana flower purchase PY	14.9%	16.7%	21.8%	***P* < 0.001**
Any flower purchase among PY users^c^	60.3%	64.0%	68.8%	*P* = 0.052
Any flower purchase among DND users	82.7%	89.6%	87.3%	*P* = 0.465
Any other marijuana product purchase PY	8.0%	8.9%	13.0%	***P* = 0.003**
Any other product purchase among PY users	32.7%	34.2%	41.0%	*P* = 0.075
Any other product purchase among DND users	50.4%	54.9%	54.1%	*P* = 0.672
Ever purchased at retail store among PY users^d^	23.4%	41.4%	65.1%	***P* < 0.001**
Ever purchased at retail store among DND users	34.7%	54.7%	85.4%	***P* < 0.001**

^a^Trend test is performed fitting logistic regressions predicting each dichotomous outcome using year as a continuous predicting variable

^b^Among PY users only

^c^Marijuana purchase questions were only asked if the respondent was a PY user

^d^Including marijuana flower or marijuana products, and only asked if the respondent was a PY user. Only wave 2 data were used for 2014, excluding wave 1 data as the survey was conducted before any store was opened

Table 2 presents the sample means and standard errors for the marijuana flower and other marijuana product purchase measures across 2014–2016, and also separately by DND and non-DND users. Across years, there were only a few significant trends observed, all among non-DND users including a decrease in the number of days flower was purchased and lower total grams of flower purchased. There were clear differences between DND and non-DND users in purchasing behaviors. Compared to non-DND users, the flower purchasing days of DND users were about 2–4 times greater and the usual grams of marijuana flower purchased was about double. The mean price of marijuana flower paid by DND users was $5.2 per gram less than for non-DND users in 2014 with this gap shrinking to about $2.6 in 2016. DND users also purchased other marijuana products more frequently and spent more on each purchase, with the exception of 2016 where the mean usual expenditure spent on other marijuana products was similar to non-DND users ($36.3 vs $38.7).

**Table 2:** Means (SE) for marijuana flower and other marijuana products purchase quantity, frequency, and expenditure for Washington state 2014–2016

	**2014**	**2015**	**2016**	**Trend test**
# days marijuana flower purchased PY^a^	25.8 (2.3)	20.8 (2.5)	20.2 (2.2)	*P* = 0.098
- For daily/nearly daily (DND) users	33.7 (3.2)	31.5 (4.0)	34.6 (3.8)	*P* = 0.838
- For non-DND users	15.4 (3.0)	8.0 (1.0)	8.3 (1.0)	***P* = 0.029**
Grams of marijuana flower usually purchased^b^	13.7 (4.7)	9.8 (1.6)	7.3 (1.0)	*P* = 0.158
- For DND users	17.7 (7.7)	13.1 (2.6)	10.1 (1.9)	*P* = 0.327
- For non-DND users	8.3 (3.6)	5.7 (1.3)	4.9 (0.9)	*P* = 0.340
Total grams marijuana flower purchased PY^b^	202 (27)	165 (27)	186 (68)	*P* = 0.882
- For DND users	294 (41)	270 (44)	374 (142)	*P* = 0.581
- For non-DND users	78.0 (24.1)	36.8 (8.2)	29.6 (4.6)	***P* = 0.044**
$ per gram for usual marijuana flower purchase^c^	11.5 (0.8)	13.6 (0.9)	11.5 (0.5)	*P* = 0.786
- For DND users	9.3 (1.0)	11.7 (1.2)	10.1 (0.5)	*P* = 0.543
- For non-DND users	14.5 (1.2)	16.0 (1.3)	12.7 (0.8)	*P* = 0.120
# days other marijuana product purchased PY^d^	17.0 (3.4)	15.2 (2.7)	11.2 (1.7)	*P* = 0.098
- For DND users	22.5 (5.2)	21.4 (4.0)	15.3 (3.2)	*P* = 0.225
- For non-DND users	7.9 (1.5)	5.0 (0.9)	7.6 (1.3)	*P* = 0.877
$ usually spent purchasing other marijuana products^e^	33.2 (5.6)	41.3 (7.8)	37.5 (4.9)	*P* = 0.668
- For DND users	38.4 (8.1)	48.8 (11.6)	36.3 (4.7)	*P* = 0.730
- For non-DND users	22.7 (3.7)	27.5 (3.7)	38.7 (8.5)	*P* = 0.100

^a^For PY marijuana flower purchasers (*n* = 710)

^b^For PY marijuana flower purchasers excluding 16 respondents reporting neither valid usual quantity nor valid expenditure (*n* = 694)

^c^For PY marijuana flower purchasers who reported both valid usual quantity and expenditure (*n* = 597)

^d^For PY purchasers of other marijuana product (*n* = 391)

^e^For PY purchasers of other marijuana product excluding 49 respondents who did not report valid expenditure on other marijuana products (*n* = 342)

Table 3 shows the total market size for marijuana flower and other marijuana products in Washington across 2014–2016 and by user frequency groups. We estimate that the marijuana flower market size in Washington was 158 metric tons (MT) in 2014 and increased to 222 MT in 2016. All DND users purchased 130 MT in 2014, increasing to 203 MT in 2016, representing 82–91% of total flower sales in quantity across the 3 years (88% for the three years combined). Total expenditure on marijuana flower was about $1.23 billion ($0.97 billion by DND users) in 2014 and increased to $1.70 billion ($1.52 billion by DND users) in 2016. The proportion of total expenditure on flower purchased by DND users ranged from 79 to 89% (86% for three years combined). The mean market price of marijuana flower was $7.81 per gram in 2014, increased to $8.68 in 2015, and dropped back to $7.65 per gram in 2016. Note that the market price reported in Table 3, derived by dividing the total market dollar expenditures by total market quantity, was lower than the average price among purchasers reported in Table 2, which was the sample mean. The total expenditure on other marijuana products in Washington was estimated at $0.44 billion ($0.41 billion by DND users, 93%) in 2014, declining to $0.33 billion ($0.22 billion by DND users, 67%) in 2016, with purchases by DND users representing 84% of total expenditure for three years combined. Taken together, the results show that the marijuana market in Washington has grown substantially since retail stores began to open in 2014, by 40.5% for marijuana flower quantity (158 to 222 MT) and 21.5% for total expenditures, including both flower and other marijuana products ($1.67 to $2.03 billion). Several sensitivity analyses were performed for market size estimation. First, when accounting for potential overestimation of flower purchasing frequency, the re-estimated flower purchasing quantity was 145, 135, and 218 MT across 2014–2016, while the re-estimated flower expenditure was 1.15, 1.17, and 1.67 billion dollars across the three years. For the market size of other marijuana products, when usual expenditure was estimated by taking the average for multiple products, the re-estimated total expenditure was 0.38, 0.30, and 0.24 billion dollars for years 2014–2016; when taking the summation across multiple products, the re-estimated total expenditure was 0.50, 0.48, and 0.41 billion dollars across the years (not shown).

**Table 3:** Total market size estimates (95% CIs) for marijuana flower quantity and expenditure and for other marijuana product expenditure for Washington state from 2014 to 2016

	**2014**	**2015**	**2016**
Total marijuana flower purchased (metric tons)^a^	158 (106, 209)	148 (93, 204)	222 (58, 387)
- For daily/nearly daily (DND) users	130 (82, 179)	133 (79, 187)	203 (39, 367)
- Dor non-DND users	27.0 (10.3, 43.7)	15.1 (7.9, 22.2)	19.3 (12.1, 26.5)
Total expenditure on marijuana flower (billion $)^a^	1.23 (0.87, 1.59)	1.29 (0.83, 1.75)	1.70 (0.47, 2.93)
- For DND users	0.97 (0.65, 1.28)	1.15 (0.70, 1.60)	1.52 (0.29, 2.74)
- For non-DND users	0.26 (0.10, 0.43)	0.14 (0.08, 0.20)	0.18 (0.12, 0.25)
$ per gram of usual marijuana flower ^a^	$7.81	$8.68	$7.65
- For DND users	$7.41	$8.61	$7.48
- For non-DND users	$9.77	$9.35	$9.53
Total expenditure other marijuana products (billion $)^b^	0.44 (0.09, 0.80)	0.39 (0.17, 0.60)	0.33 (0.17, 0.49)
- For DND users	0.41 (0.05, 0.76)	0.35 (0.14, 0.57)	0.22 (0.07, 0.38)
- For non-DND users	0.04 (0.02, 0.05)	0.03 (0.01, 0.06)	0.10 (0.06, 0.15)

^a^For PY marijuana flower purchasers (*n* = 710). Among them, 16 respondents reported neither valid usual quantity nor valid expenditure and the measures were imputed using sample median

^b^For PY purchasers of other marijuana product (*n* = 391). Among them, 49 respondents whose expenditure on other marijuana products were missing and the measure was imputed using sample median

Finally, using the 2016 data only, PY total expenditure on marijuana flower based on store sales (including sales from both legal retail stores and medical dispensaries) was estimated at $1.24 billion, representing 73% of the $1.70 billion total expenditure from the 2016 PSW surveys ($1.14 billion by DND users and $0.11 billion by non-DND users). Likewise, the total market size for marijuana flower quantity from store sales was estimated at 153 MT, 69% of the 222 MT total quantity based on the 2016 survey (143 and 10.3 MT by DND and non-DND users, respectively). The remaining 27% of expenditures and 31% of quantity purchased from “other sources” was the estimated market share of the illegal market.

## Discussion

These 2014–2016 market size estimates and analyses of purchasing behaviors for the state of Washington (age 18 +) build on the small number of population studies addressing marijuana buying and use in a legalized market. This study is the first to provide estimates directly from a representative sample of a state during the early years of retail store openings. Surveys are an important tool for estimating and tracking legal and illegal marijuana use and appear to be more accurate after legalization (Kerr et al. 2018). Furthermore, marijuana purchasing and use appear to be very concentrated among the heaviest users, like alcohol and many other products.

Our results are reasonably similar to prior studies utilizing different data. An analysis by Caulkins and colleagues of the Washington market used the 2015–2016 NSDUH surveys and RAND’s 2013 survey of Washington cannabis users to estimate market size and found a total market size of 208 MT for Washington in 2016, worth $1.66 billion (Caulkins et al. 2019). These amounts are comparable to, but lower than, our estimates of $2.0 billion on all products and 222 metric tons of marijuana flower. RAND estimates for 2016–2017 were higher at 252 MT for total market size reflecting the increased use seen for 2016 in the PSW survey and for 2016–2017 in the NSDUH survey (Kilmer et al. 2019). It is notable that population estimates of total alcohol consumption from survey use measures are typically 40–60% of sales figures (Nelson et al. 2010; Kerr, et al. 2010), suggesting that cannabis market estimates based on surveys are also likely to be conservative. Our estimates for 2016 also indicate that about 31% of the marijuana sold in Washington came from outside the legal retail system, indicating that the legal system provided the most, but still far from all, of the total market. Comparison of the prices paid for flower in the surveys with those in the Washington track-and-trace system show that they became more similar over time. The system prices were $4.79 higher per gram in 2014, but declined over time and were slightly lower in 2016 at $7.20 per gram compared to $7.65 in the PSW surveys (Davenport 2021). The declining system prices were due to both increased supply of cannabis and the 2015 tax reduction. Total expenditure in 2016 in the system was $969 million compared to $2.03 billion in the surveys, suggesting that about $411 million was spent in dispensaries, which remained open until June of 2016, and $620 million was spent in the illicit market.

A key finding was that daily and near daily (DND) users bought 91% of the marijuana flower in 2016 and spent 89% of the dollars spent on other marijuana products. The high proportion of cannabis sold to DND users also suggests that they are the main providers of cannabis to the 25% of users who did not purchase in the past year. Prior studies have also highlighted the importance of DND users: A US study (Davenport and Caulkins 2016) found that they used 77% of the cannabis in 2012–2013. An Australian study also found daily users accounted for 85% of the cannabis in 2016 (Chan and Hall 2020). These findings indicate that the cannabis market is largely driven by DND users and that the concentration of purchasing in this group does not decline, and perhaps increases, after legalization. In our results there was a reduction in the proportion of past year users who are DND from about 41% to 36% in 2016 as the prevalence of past year use increased, indicating that most new users had lower frequencies.

There are a number of limitations to this study including the use of self-reported purchasing behavior, which may be mis-remembered or under-reported due to social desirability bias and concerns about reporting illegal activities for illicit market purchases (Kerr et al. 2018). There may also be under-reporting due to non-response if heavier cannabis users/purchasers were less likely to participate in the surveys as is the case for alcohol (Tolonen et al. 2019). While this is likely also the case for stronger drugs such as heroin it is not clear whether this would apply to cannabis users. Our analyses also required some strong assumptions regarding the interpretation of reported frequency of purchases and the relationships between the most recent purchase and other purchases. The use of recent purchase questions is supported by a study that included multiple purchases and found that the most recent was representative of other purchases (Bond et al. 2014).

Surveys of purchasing behaviors offer a unique and important perspective on marijuana markets in states with legal use enabling tracking of the shift from illicit to legal markets and the expansion of purchasing among users. Surveys are also needed to understand purchaser characteristics and the concentration of activities among certain types of users/purchasers. There is a need for more detailed monitoring surveys such as ours to track behaviors in states adopting retail marijuana markets. These should include more details on products and purchasing patterns than was possible in our PSW surveys, which were designed primarily to capture alcohol use and purchasing. Other marijuana products, particularly concentrates, vape pens, and edibles, have become more popular in recent years increasing the importance of more detailed assessment and tracking (Schauer et al. 2016; Carlini et al. 2020).

## Conclusions

This study utilized the 2014–2016 PSW surveys to estimate the total market size for cannabis flower and other cannabis products in each year with individually-reported data from within the surveys only, with results similar to those from prior estimates that used multiple sources. The cannabis market was shown to increase over time, particularly in 2016 when the full complement of retail stores were open with an estimated 70% of cannabis purchased legally in that year. Detailed analyses of cannabis purchasing behaviors highlight the importance of DND users who accounted for most of the purchases in each year. These results emphasize the importance of focusing prevention and intervention efforts on 10–11% of the population who are DND users who not only account for the majority of cannabis use but also likely provide most of the cannabis to the 25% of past-year users who did not purchase any themselves.

## Data Availability

Data are not currently publicly available as the study is ongoing.

## Abbreviations

NSDUH: National Survey on Drug Use and Health
DND: Daily or nearly daily
MT: Metric tons
PY: Past year
RDD: Random digit dialed
